# Plant–Endophyte Cross-Talk in *Origanum heracleoticum* L. In Vitro Axenic Culture: Endosphere-Driven Bacterial Interactions and Plant Metabolic Responses

**DOI:** 10.3390/microorganisms14071497

**Published:** 2026-07-08

**Authors:** Giulia Semenzato, Sara Barberini, Felicia Menicucci, Giulia Atzori, Cecilia Brunetti, Giovanni Marino, Valeria Palchetti, Renato Fani, Mauro Centritto, Giovanni Emiliani

**Affiliations:** 1 Institute for Sustainable Plant Protection, National Research Council of Italy, 50019 Sesto Fiorentino, Florence, Italy; giuliasemenzato@cnr.it (G.S.); felicia.menicucci@cnr.it (F.M.); giulia.atzori@cnr.it (G.A.); cecilia.brunetti@cnr.it (C.B.); giovanni.marino@cnr.it (G.M.); valeriapalchetti@cnr.it (V.P.); mauro.centritto@cnr.it (M.C.); giovanni.emiliani@cnr.it (G.E.); 2 Department of Biology, University of Florence, 50019 Sesto Fiorentino, Florence, Italy

**Keywords:** endophytic microbial ecology, oregano, beneficial microorganisms, *Bacillus*, *Pseudomonas*, rosmarinic acid, alpha-humulene

## Abstract

*Origanum* L. (Lamiaceae) is a commercially important medicinal and aromatic plant genus worldwide. Endophytic bacterial communities are recognized for promoting plant growth and physiology, although their interactions with host metabolism remain insufficiently understood. In this work, an in vitro model of axenic *Origanum heracleoticum* plants was established to investigate the relationship between endophytic bacteria and their tissue of origin. Specifically, we evaluated the adaptation of two strains, *Bacillus* sp. OHL2 and *Pseudomonas* sp. OHS18, and the potential role of *Bacillus* sp. OHL2 in modulating plant physiology and secondary metabolism. Bacterial inoculation and re-isolation highlighted niche-specific adaptation and possible co-evolution within the host, suggesting an active role of the plant in regulating bacterial colonization within the endosphere. Inoculation with *Bacillus* sp. OHL2 significantly enhanced photosynthetic rate, leaf area, dry weight, and chlorophyll content. No substantial overall changes in secondary metabolism were detected. Rosmarinic acid was the predominant phenolic, while monoterpenes dominated, with carvacrol dominant. A significant tissue-by-inoculation interaction was observed for α-humulene, which decreased in leaves of inoculated plants. Overall, the in vitro system provides a valuable platform to study plant–endophyte interactions and bacterial mechanisms underlying the stimulation of plant growth and metabolic responses.

## 1. Introduction

*Origanum* (L.) (Lamiaceae) is one of the most commercially valuable medicinal and aromatic plants worldwide, used both as a culinary herb and for its essential oil in flavoring, dietary supplements, cosmetics, and aromatherapy [1]. Moreover, due to its remarkable antimicrobial and antioxidant properties, oregano essential oil holds significant potential for biotechnological, industrial, and pharmaceutical applications [1,2].

*Origanum heracleoticum*, a common subspecies of *O. vulgare* (subsp. *viridulum* (Martrin-Donos) Nyman or subsp. *viride* (Boissier) Hayek), is widely distributed in Sicily (Italy), representing an essential component of local gastronomy [3]. The increasing market demand for high-quality medicinal and aromatic plants has led to a renewed interest in the cultivation of local oregano biotypes in southern Italy, a trend that preserves traditional agricultural practices and provides economic opportunities for local farmers [3]. The unique pedoclimatic conditions of this region contributed to the development of particularly intense aromatic profiles. Notably, *O. heracleoticum* is highly valued for its strong aroma, which is attributed to the abundance of essential oil-bearing glands on its leaves, calices, and bracts [4,5]. While chemical traits are largely influenced by genetic and environmental factors, increasing attention has been given to the role of plant-associated microbiota in modulating plants’ physiological status, metabolic functioning, and overall performance. Plants have coevolved with their associated microbial communities through signaling interactions of various molecular natures [6,7] and, more recently, the endophytic bacterial microbiota of medicinal and aromatic plants has gained attention for its role in promoting plant growth and performance and modulating its secondary metabolism [8].

A critical aspect of host–endophyte relationship analysis is understanding the specificity of these interactions within each plant inner tissue [9]. Notably, it was often evidenced that the endophytic communities within different anatomical parts of the same plant display significant variation in genera and species richness [10,11]. The plant itself seems to play an active role in selecting specific bacteria, suggesting an interaction in which particular endophytes are favored during the colonization process for their contribution to tissue-specific metabolism and physiological functioning [12]. This selection hints at a functional specificity, where the activities performed by endophytes—such as nitrogen fixation, hormone production, protection against pathogens [13,14,15,16]—are tailored to the needs of each plant compartment [17]. For example, leaf-associated endophytes are believed to maintain a positive carbon balance under stress by modulating mesophyll CO_2_:O_2_ ratios, reducing stomatal conductance and transpiration, and improving intrinsic water use efficiency [18,19].

Beyond host-derived factors, microbe–microbe interactions play a key role in shaping plant colonization and microbial community assembly [20], and synthetic communities may provide useful systems to study these interactions under controlled conditions. Recent studies on *Arabidopsis thaliana* leaf-associated bacteria have shown that negative interactions dominate the phyllosphere, with ~90% of co-inoculations reducing the population density of at least one partner [21], largely due to metabolic niche overlap and competition for limiting resources, particularly sugars [22].

A further level of complexity is introduced by the diverse repertoire of secondary metabolites in medicinal and aromatic plants. Differences in the structure and composition of endophytic bacterial communities may be influenced by the presence of distinct bioactive compounds with antimicrobial activity across plant species and compartments [12,23,24]. Notably, essential oil content in *O. vulgare* plants was correlated with the distribution of lactic acid bacterial (LAB) endophytes, supporting the hypothesis that LABs, such as *Lactobacillus plantarum*, persist as endophytes due to their tolerance to oregano’s essential oil components [25]. The selective pressures exerted by the presence of bioactive compounds in the plant’s inner tissues not only shape the bacterial communities but also suggest a degree of metabolic adaptation, where certain endophytes have evolved to resist or even utilize essential oils as carbon sources [26]. Moreover, the bacterial contribution to the shaping of plant-produced bioactive compounds cannot be a priori excluded [27,28]. Secondary metabolites in *Atractylodes lancea* and *Panax quinquefolius* were found to correlate with specific endophytic microbial taxa. These relationships were confirmed by inoculation experiments showing that introducing selected bacterial strains alters metabolite composition, highlighting the role of endophytes in modulating the biosynthesis of pharmacologically relevant compounds [29,30]. Further, in *Echinacea purpurea*, endophytes preferentially re-colonized their original plant organ, and increased alkamide levels and valine decarboxylase gene expression, suggesting a role in alkamide biosynthesis [31,32,33].

In a previous study on the cultivable endophytic microbiota of three different *Origanum* species, it was revealed that different aerial compartments of the same plant—namely stems, leaves, and flowers—shared very few culturable bacterial strains [23]. In the case of *O. heracleoticum*, this pattern was associated with the selective recruitment of bacterial taxa and functions, microbial competition, and the metabolic composition of individual plant compartments [34]. Based on these findings and within the framework of microbiome compartmentalization, we hypothesized that endophytic strains exhibit distinct compartment-associated colonization patterns reflecting adaptation to specific host tissues, and that selected endophytes can influence specific plant organ metabolism. To test this hypothesis, in this work, we established an in vitro model of axenic *O. heracleoticum* plants. Specifically, we investigated the colonization patterns of endophytic strains previously isolated from different plant compartments through both single- and co-inoculation experiments, and evaluated the ability of a selected strain to influence the physiology and secondary metabolism of specific plant compartments.

## 2. Materials and Methods

### 2.1. Bacterial Strains and Growth Conditions

Two bacterial endophytic strains, namely *Bacillus* sp. OHL2 and *Pseudomonas* sp. OHS18, were selected from a previously established collection, based on their adaptation to well-defined compartments of *O. heracleoticum* (leaves or stems) and their taxonomic affiliation [23]. Specifically, in *O. heracleoticum* plants, all cultivable isolated *Pseudomonas* strains were found exclusively in the stems, while *Bacillus* ones were mostly represented in both flowers and leaves. However, strain OHL2 was (only) found in leaves, as revealed by a RAPD analysis. The two strains exhibited distinct colony morphologies, facilitating their identification when co-cultured on TSA medium. Furthermore, cross-streaking tests confirmed the absence of mutual inhibition, enabling their co-inoculation.

Bacteria were cultured on Tryptic Soy Agar medium (TSA, Oxoid, Hampshire, UK) at 30 °C for 48 h.

### 2.2. Bacterial Genome Analysis

A single colony of each strain was inoculated into 10 mL of fresh TSB (Biolife, Milan, Italy) and incubated overnight (30 °C, 130 rpm). Cells were pelleted using centrifugation, and genomic DNA was extracted using the PowerLyzer PowerSoil DNA Isolation Kit (MO BIO Laboratories, Inc., Carlsbad, CA, USA) following the manufacturer’s protocol with the modifications described in Semenzato et al. (2022) [35].

Genome sequencing was performed with a PCR-free approach, following the native barcoding genomic DNA protocol by Oxford Nanopore Technologies (ONT) (version NBE_9065_v109_revY_14Aug2019). Library preparation, sequencing, basecalling, and demultiplexing were performed according to Semenzato et al., 2022 [35].

De novo assembly was generated by Canu v2.1.1 [36]. Contig quality was evaluated using QUAST v.5.2.0 [37]. Average Nucleotide Identity (ANI) was performed using FastANI v.1.3 [38]. Reference genomes from *Bacillus* and *Pseudomonas* were downloaded from the National Center for Biotechnology Information (NCBI) “assembly” database and used for ANI analysis, in a Galaxy environment (https://usegalaxy.eu, accessed on 19 September 2022). Whole-genome-based taxonomic classification was performed using the Type (Strain) Genome Server (TYGS; https://tygs.dsmz.de, accessed on 30 May 2025), with results provided on 26 June 2025.

Genome annotation was performed using Prokka v.1.14.6 [39] and the NCBI Prokaryotic Genome Annotation Pipeline (PGAP), v.6.2 (https://www.ncbi.nlm.nih.gov/genome/annotation_prok/, accessed on 14 October 2025). Bacterial PGP traits were identified using the PGPT-Pred tool (blastp + hmmer, strict mode) within the PLaBAse server (Patz et al., 2021), and annotated using the blastp  +  hmmer (strict mode), mapping against the plant growth-promoting traits (PGPT) ontology, within the “PGPT-Pred” tool provided by the PLaBAse server [40]. Heatmaps were generated in R v.4.5.2, using the ‘ggplot2’ package.

### 2.3. Determination of the Bacterial Growth Curve

Single colonies of *Bacillus* sp. OHL2 and *Pseudomonas* sp. OHS18 were separately inoculated in 5 mL of TSB and incubated under shaking at 27 °C for eighteen hours. Each bacterial liquid culture was then diluted in TSB to reach a final optical density (OD, 600 nm) of 0.01. Cells were incubated at 27 °C, and the OD_600_ was measured every hour for two days using the Infinite M Flex microplates reader (Tecan, Mannedorf, Switzerland). Each curve was performed in triplicate. Curve parameters and graphs were obtained in the R environment using ‘growthcurver’ and ‘ggplot2’ libraries [41,42].

### 2.4. Mutual Synergistic or Competitive Interactions of Endophytic Strains

*Bacillus* sp. OHL2 and *Pseudomonas* sp. OHS18 were grown on Ayers Mineral Salt Medium (NH_4_H_2_PO_4_, 1 gr/L; KCl, 0.2 gr/L; MgSO_4_·7H_2_O, 0.2 gr/L; pH = 7.2) amended with 0.2% glucose for 48 h at 27 °C. Cultures were centrifuged at 8000 rpm for 10 min, and the supernatants were sterilized by filtration (Millipore 0.2 µm filters, Darmstadt, Germany). Two mL of the culture filtrates from one strain culture was mixed with 2 mL of Ayers medium supplemented with 0.1% glucose and inoculated with 30 µL of a bacterial suspension (OD_600_ = 0.1) of the other strain. These new cultures were incubated at 27 °C for 2 and 5 days under shaking. The OD_600_ of all the obtained culture combinations was measured and compared with that reached by each bacterium grown in Ayers medium + 0.1% glucose alone. The experiment was performed in triplicate.

### 2.5. Seed Sterilization and Plant Micropropagation Procedure

**Figure 1:**
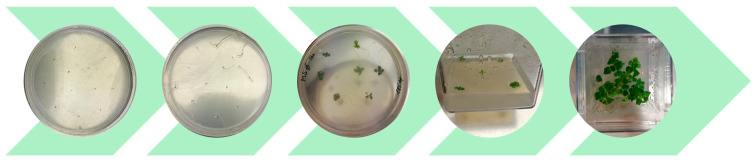
In vitro propagation of *O. heracleoticum*. From left to right: sterilized seeds placed in water agar plates; germinated seeds; *O. heracleoticum* shoots placed in MS medium; *O. heracleoticum* shoots in Magenta vessel containing MS + BA 0.2 mg/L; *O. heracleoticum* plantlets in MS + BA 0.2 mg/L prior to the inoculation.

Clonal lines of oregano were obtained from individual seedlings germinated in vitro from a heterogeneous seed population (Figure 1), courtesy of “Il Giardino delle Erbe”, Casola Valsenio. Seeds were surface-sterilized in 70% EtOH (1 min) and 1% NaClO (15 min), rinsed three times with sterile water, and placed on water-agar plates for three days in the dark. Then seeds were transferred to light, in MS medium [43] supplemented with 3% sucrose. Each seedling generated one clonal line, which was micropropagated in MS supplemented with Benzylaminopurine (BA, 1 mg/L) in Magenta GA-7^®^ vessels. The presence of cultivable endophytes inside the host tissues was checked to validate the sterility of the model system: shoots and roots from three plants were homogenated in 0.9% NaCl (500 µL) and 100 µL aliquots were plated on TSA. Plates were monitored for seven days at 30 °C, and no bacterial colonies were isolated from the plantlets’ tissues.

Clonal lines OH-CL 3 and OH-CL 4, showing comparable morphology and growth rate under standard micropropagation conditions, were selected for two independent experiments.

For isolation assays, five shoots per Magenta vessel were grown on MS + 0.2 mg/L BA (three vessels per condition). For physiological and metabolite analyses, six shoots per Weck jar were prepared (five jars per condition). All cultures were sealed with parafilm and maintained at 24 °C under a 16 h photoperiod.

### 2.6. Plantlet Inoculation

The two selected bacterial endophytes were retrieved from their glycerol stock and allowed to grow on TSA plates at 30 °C for 48 h. A single colony of each strain was separately inoculated in 10 mL of Tryptic Soy Broth (TSB, Oxoid, UK) and incubated for eighteen hours at 30 °C at 130 rpm. The next day, bacterial suspensions were adjusted to approximately 10^8^ CFU/mL (OD_600_ = 1) in a final volume of 5 mL of TSB. The suspensions were centrifuged at 4500 rpm for 15 min at room temperature, the supernatant was removed, and the pellet was resuspended in a corresponding volume of saline solution. The OD_600_ was normalized to 0.01 (approximately 10^6^ CFU/mL), as higher concentrations caused plant damage within 14 days post-inoculation.

**Figure 2:**
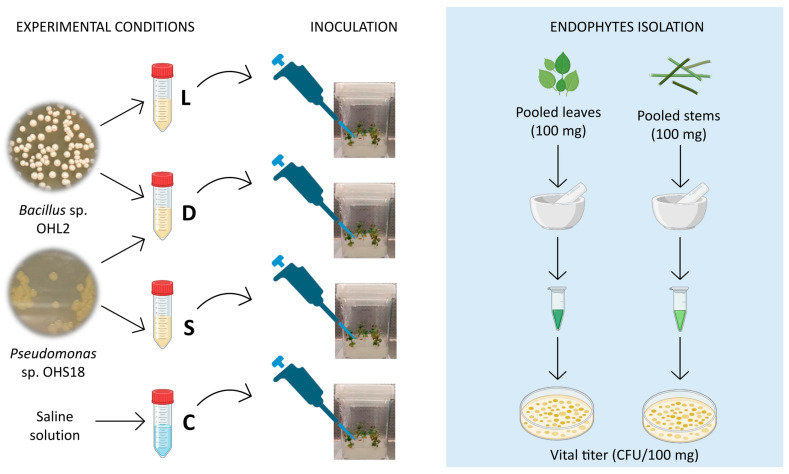
Workflow of the plantlet inoculation and the endophyte isolation phases. Bacterial suspensions (L, S, D) were adjusted to an OD = 0.01 in saline solution, and 50 μL were used to inoculate each of the five plantlets cultivated in a Magenta vessel. Additionally, 50 μL of saline solution were used to inoculate control plants (C). After the incubation time, 100 mg of leaves and stems were collected separately after sterilizing and pooling the plants. Each tissue was homogenized in a sterile mortar with 500 μL of saline solution, which was then diluted and plated to obtain the vital titer.

Four experimental conditions were set up (Figure 2): (i) bacterial suspension containing *Bacillus* sp. OHL2 (L group), (ii) *Pseudomonas* sp. OHS18 (S group), (iii) both strains (D group), and (iv) only saline solution as the control (C group). Serial dilutions of the four suspensions were plated on TSA plates to obtain the viable titer (CFU/mL, Table 1).

The bacterial inoculum (or saline solution) was provided in the MS medium at the collar of each plant. In detail, for a first set-up experiment, five 14-day-old *O. heracleoticum* shoots (clonal line OH-CL 4) contained in a Magenta vessel (representing one replicate) were each inoculated with 50 μL of the bacterial suspensions or sterile saline solution (L, S, D, or C), in triplicate. Plantlets were then incubated in the growth chamber for 14 and 28 days.

A second inoculation experiment was carried out using five 14-day-old *O. heracleoticum* shoots (clonal line OH-CL 3) contained in a Magenta vessel (in three replicates) and six plantlets cultivated in a Wack glass jar (in five replicates). Similarly, each shoot was inoculated with 50 μL of the bacterial suspensions or saline solution. Plantlets were then incubated in the growth chamber for 21 days.

### 2.7. Endophyte Isolation

After incubation, endophytes were isolated from the plantlets grown in Magenta vessels (Figure 2). Plantlets were cut at the collar, and the upper sections were surface sterilized in 1% (*v*/*v*) NaClO for 2 min and 30 s, then rinsed three times with sterile distilled water. Sterilization efficacy was assessed by plating final rinse water to ensure that recovered bacteria originated only from internal tissues. To obtain sufficient biomass, 5 plantlets were pooled per replicate to obtain at least 100 mg of leaves and stems. The presence of inoculated endophytes in individual plantlets had been previously verified. For each replicate, 100 mg of tissue was homogenized in a sterile mortar using 500 μL of saline solution. Homogenates were centrifuged at 0.8 rpm for 15 min, and supernatants were serially diluted and plated on TSA. Colony-forming units (CFU/100 mg) were recorded after two days of incubation at 30 °C. Because leaf and stem samples originated from the same plant, the two measurements are not statistically independent; the tissue effect was therefore assessed using the Wilcoxon signed-rank test. The 14-day, 28-day, and 21-day timepoints were analyzed separately, each as an independent tissue comparison. *p*-values were adjusted for multiple comparisons across the two timepoints using the Benjamini–Hochberg FDR procedure.

### 2.8. Plant Physiology and Morphology

Five Weck jars per treatment, each containing six plantlets inoculated with either the bacterial suspension (L) or with saline solution (C), were used to assess the effect of *Bacillus* sp. OHL2 inoculation on plant photosynthetic rate (A). An infrared gas analyzer (Ciras 3, PP Systems, Amesbury, MA, USA) was connected to each sealed jar by inserting the inlet and outlet tubes through the jar cap. During measurements, jars were exposed to an externally applied photosynthetic photon flux density (PPFD) of 600 µmol m^2^ s^−1^, and to a CO_2_ concentration of 420 ppm supplied at a constant flow rate of 500 cc min^−1^. Once air exchange in the jar was completed and the CO_2_ concentration reached a steady state, the CO_2_ level at the jar outlet was recorded. Following the removal of the plantlets, measurements were repeated using jars containing only agar substrate to determine background CO_2_ exchange. Net photosynthesis per jar was subsequently calculated as the difference between outlet CO_2_ measured in the presence of plantlets and that measured with agar substrate alone (ΔCO_2_), relative to an inlet CO_2_ concentration of 420 µmol mol^−1^.

The total leaf area (LA) of the plantlets was then calculated using ImageJ software Version 1.54p [44]. The photosynthetic rate (A, µmol m^2^ s^−1^) on a leaf area basis was calculated according to the following equation:

(1) $$A=\frac{\left(∆{CO}_{2}\times \mathrm{Flow}\mathrm{rate}\right)}{LA}$$

where ΔCO_2_ is the difference in CO_2_ concentration measured in the jar with and without the plantlet, expressed in µmol mol^−1^; flow rate is the rate of air passing through the jar, expressed in mol s^−1^; LA is the total leaf area of the plantlet, expressed in m^2^.

Leaves from the six plantlets in each jar were separated and dried at 70 °C to constant weight to quantify the mean leaf dry mass and total dry biomass per jar. Total LA calculated for each jar was averaged across the five replicates to obtain the mean leaf area per jar. Leaf area (LA) per plantlet was averaged across five jar replicates to obtain mean plantlet leaf area.

### 2.9. Secondary Metabolites of O. heracleoticum In Vitro Plants

#### 2.9.1. Pigment Content

Chlorophyll a, chlorophyll b, and carotenoid concentrations were determined on three replicates per treatment according to [45]. Fresh leaf tissue (0.1 g) was collected in centrifuge tubes, frozen in liquid nitrogen, and stored at −80 °C until analysis. A tungsten bead (ø 5 mm) was added leaves were disrupted using the Tissue Lyser II system (QIAGEN, Venlo, the Netherlands, cat. no. 85,300) for 30 s at 30 Hz. Cold 100% methanol (MeOH) was added, and extracts were shaken in darkness at 4 °C for 30 min. Samples were then centrifuged at 1000 rpm for 10 min, and absorbance of the supernatant was recorded at 665, 652, and 470 nm using a Tecan Infinite 200 spectrophotometer (Männedorf, Switzerland).

#### 2.9.2. Phenylpropanoid Content

Five Weck jars per treatment, each containing six plantlets inoculated with either the *Bacillus* sp. OHL2 suspension (L) or saline solution (C), were used to assess effects on polyphenol production. Before the biochemical analyses, stems and leaves were separated, weighed, frozen with liquid nitrogen and lyophilized. For each replicate, 3–40 mg of dried tissue was extracted with 3 × 0.5 mL of ethanol 75% solution (pH 2.5, formic acid) using an ultrasonic bath at 4 °C, for 20 min. Extracts were centrifuged at 14.800 rpm for 5 min, and the supernatants were partitioned with 4 × 0.5 mL of n-hexane to remove lipophilic compounds. The hydroethanolic phase was dried in a vacuum concentrator, and resuspended in 100 µL of MeOH: Milli-QH2O solution (1:1 *v*/*v*, pH 2.5).

Polyphenol identification and quantification were obtained using an LC-QTOF (Agilent 6530C, Agilent Technologies SpA, Milan, Italy) equipped with a quadrupole time-of-flight mass spectrometer operating in the electrospray ionization (Dual AJS ESI, Agilent Technologies SpA, Milan, Italy) in negative mode, coupled to a diode array detector (DAD). The applied ESI parameters were as follows: capillary voltage 4000 V; fragmentor 180 V; skimmer 60 V; OCT 1 RF Vpp 750 V; pressure of nebulizer 20 psi; drying gas temperature 325 °C; sheath gas temperature 400 °C. Separation was performed using an Agilent Poroshell 120 EC-C18 column (2.7 μm) (Agilent Technologies SpA, Milan, Italy), with a matching pre-column, applying a 71 min gradient of water acidified with 0.1% formic acid (A) and acetonitrile acidified with 0.1% formic acid (B) combined as follows: (97% A, 3% B for one minute; reaching 70% A, 30% B at minute 53; reaching 3% A, 97% B at minute 60; holding at 3% A, 97% B until minute 62; reaching 97% A, 3% B at minute 63; holding at 97% A, 3% B until minute 71). Column temperature was 30 °C, flow rate 0.30 mL min^−1^, and injection volume 0.2 μL. Quantification was performed in DAD at 330 nm using a six-point calibration curve of the rosmarinic acid standard. Results are expressed as mean ± standard error (mg/g DW) of five replicates per treatment.

#### 2.9.3. Terpene Content

From each jar, stems and leaves of *O. heracleoticum* were separated (100–200 mg fresh weight, each) and placed in 10 mL glass vials. Heptane (0.5–1 mL) with tridecane was added for the terpene extraction. Then, each vial was vortex-mixed, sonicated for 30 min (3 cycles of 10 min each), and kept on overnight rotary agitation at 25 °C. After centrifugation at 4000 rpm for 10 min, the extract was collected for gas chromatography-mass spectrometry (GS-MS) analysis. An Agilent model 5977C GC/MSD Gas Chromatograph (GC) system equipped with a Mass Selective Detector (MSD) with EI ionization was employed (Agilent Technologies SpA, Milan, Italy). One microliter of liquid extract in heptane was injected into an injector operating in split mode (1:10), and helium was used as the carrier gas with a pressure of 15.8 psi (flow of 1 mL min^−1^). The chromatographic settings were as follows: injector in split mode set at 250 °C, DB-WAX 60 m, 0.25 mm i.d., 0.5 μm film (Agilent J&W DB-WAX Ultra Inert column; Agilent Technologies SpA, Milan, Italy). The oven temperature program was as follows: initial temperature 35 °C for 2 min, then 5 °C min^−1^ until 200 °C, hold time 1 min, then 20 °C min^−1^ until 250 °C, then 5 °C min^−1^ until 240 °C, hold time 5 min. The mass spectrometer was operating with an electron ionization of 70 eV, in scan mode in the *m*/*z* range 16–330, at three scans sec^−1^. Data were acquired and analyzed using Agilent MassHunter software (v. 10.0). The deconvoluted peak spectra, obtained by Agilent Masshunter software, were matched against the NIST 20 spectral library for tentative identification. The compound validation was also obtained by injection of authentic standards to confirm the identification. Each monoterpene and sesquiterpene compound was expressed as a percentage of the total monoterpene and sesquiterpene fractions, respectively.

### 2.10. Statistical Analysis

Statistical differences in bacterial growth curve parameters and in data describing synergistic or competitive interactions were assessed using Student’s *t*-test in the R environment.

Differences in rosmarinic acid and terpene contents were analyzed using a linear mixed-effects model with Restricted Maximum Likelihood (REML) estimation, with statistical significance set at *p* < 0.05. Terpene contents were expressed as percentages of the total terpene content, and prior to statistical analysis, percentage data were converted to proportions (values ranging from 0 to 1) and transformed using the arcsine square-root transformation to meet the assumptions of normality and homogeneity of variance. The analyses were performed using GraphPad Prism 10.1.

## 3. Results

### 3.1. Genomic Analysis

*Bacillus* sp. OHL2 embeds a single contig with an overall length of 3,816,237 bp and a GC content of 41.58%. The genome was annotated using the NCBI GenBank annotation pipeline, revealing the presence of 4075 genes, of which 2824 are coding genes, 27 are rRNAs, 85 are tRNAs, and 4 are ncRNAs. The complete genome sequence is available in GenBank under the accession number JBSXQE000000000. *Pseudomonas* sp. OHS18 embeds a single contig of 5,228,754 bp with a GC content of 63.35%. A total of 4985 genes were annotated, of which 3834 are coding genes, 16 are rRNAs, 64 are tRNAs, and 4 are ncRNAs. The complete genome sequence is available in GenBank under the accession number CP182111.

Average Nucleotide Identity (ANI) analysis revealed a 98.1761% identity between *Bacillus* sp. OHL2 and *Bacillus safensis* strain PRO114 (GenBank accession GCA_029536915.2), whereas no ANI value exceeding 95% was observed for *Pseudomonas* sp. OHS18. The digital DNA–DNA hybridization (dDDH) analysis confirmed strain OHL2 affiliation to *B. safensis* species, while strain OHS18 showed dDDH values below the species threshold, suggesting it may represent a novel species.

To assess the potential plant growth-promoting (PGP) activities of the two bacteria, functional annotation was performed using PGPT-Pred via the PLaBAse server, using Prokka annotations as the input file. The functional annotation supported an endophytic, plant growth-promoting lifestyle for both bacteria, with their genomes being enriched in functions associated with plant colonization (particularly, the “plant derived substrate usage” function), biotic and abiotic stress control (“neutralizing abiotic stress”), bioremediation, biofertilization, and competitive exclusion mechanisms (Figure S1).

### 3.2. Bacterial Growth Curves and Microbial Interactions

A 48 h growth curve (OD_600_) was generated for each strain in nutrient medium (Figure S2A) to evaluate potential differences in growth rate that could influence colonization. For each strain, key growth parameters (included in the ‘growthcurver’ R package) were calculated from three biological replicates, including the maximum growth rate (*r*), area under the curve (AUC), time to half-maximum density (t_mid), and generation time (t_gen). Statistical analysis (*t*-tests) indicated that while *r* differed significantly between *Bacillus safensis* OHL2 and *Pseudomonas* sp. OHS18 (0.37 ± 0.04 and 0.16 ± 0.06, respectively; *p* = 0.008), no significant differences were observed for AUC, t_mid, and t_gen (*p* > 0.1 in all cases).

To assess possible antagonistic or synergistic interactions, each strain was grown in minimal medium supplemented or not with the other strain’s spent medium (Figure S2B). The culture filtrate of *Pseudomonas* sp. OHS18 significantly enhanced *Bacillus safensis* OHL2 growth, both after two and five days. *Bacillus safensis* OHL2 supernatant also stimulated the growth of *Pseudomonas* sp. OHS18 after two days, although the positive effect was less evident over time.

### 3.3. Bacterial Endophytes Re-Occupy Their Native Niche During Plant Colonization

**Figure 3:**
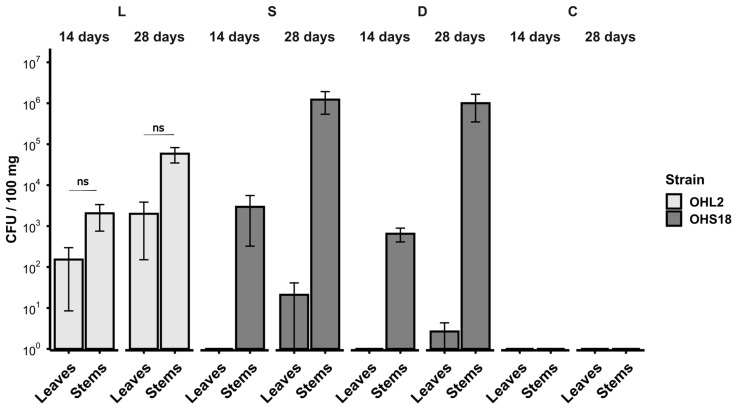
Endophyte colonization after 14 and 28 days from inoculation. Mean viable titer counts (CFU/100 mg) of the endophytic strains *Bacillus* sp. OHL2 and *Pseudomonas* sp. OHS18 obtained for each experimental group (L, S, D, and C), 14 and 28 days after the inoculation, in the stems and the leaves. Error bars represent the standard error. Statistical comparison between leaf and stem tissue at each time point was not performed for conditions S and D, where leaf samples showed absent or sporadic bacterial colonization (zero or single non-zero CFU values across biological replicates). For condition L, Wilcoxon signed-rank test was applied (*p* < 0.05). Statistical significance is indicated as follows: ns, not significant.

The two bacterial strains were used for the inoculation of in vitro axenic *O. heracleoticum* plants (clonal line CL-OH 4). Endophytes colonization was assessed at two time points to monitor the progression of bacterial establishment within the plants. For each condition, the mean vital titer of the homogenates (CFU/100 mg of plant tissue) is reported in Figure 3.

Endophyte isolation was successful after both 14 and 28 days. In general, the bacterial count detected 28 days after the inoculum was higher than the one obtained after 14 days, suggesting bacterial ability to thrive inside the plant tissues, in agreement with their endophytic lifestyle.

Data obtained for the plantlets of the L group revealed that *Bacillus safensis* OHL2 was present in both leaves and stems, with higher titers in the stems at both time points, although the difference was not statistically significant.

Concerning the S group plants, the endophytic strain *Pseudomonas* sp. OHS18 was recovered almost exclusively from the stem of the plants, with only one replicate at 28 days after the inoculum revealing its presence in the leaves (6.0 × 10^1^ CFU/100 mg). However, the bacterial concentration in the leaves was five orders of magnitude lower than in the stems, suggesting a strong niche preference for the stem compartment. Due to plant health concerns arising from the in vitro conditions, a post-infection time longer than 28 days was not feasible, preventing further evaluation of potential leaf colonization.

A peculiar situation was observed in the D group. Even though the bacterial suspension contained both endophytic strains (as demonstrated by plating the suspension to obtain its vital titer, Table 1, and although cross-streaking tests confirmed no antagonism between the two bacteria in vitro, only *Pseudomonas* sp. OHS18 was isolated from the plantlets. Similar to what was observed for the S group, the strain was almost exclusively present in the stems, with only one replicate at 28 days showing its scarce presence in the leaves (5.0 CFU/100 mg). Plating of C group samples never registered the growth of any cultivable bacteria.

### 3.4. Bacillus safensis OHL2 Inoculation Affects Plant Physiology and Secondary Metabolism

To further investigate the impact of endophytic inoculation on plant physiology and metabolism, the experiment was repeated using a different *O. heracleoticum* clonal line (OH-CL 3), evaluating endophytes colonization at a single time point (21 days post-inoculation). The absence of pre-existing culturable endophytes was confirmed throughout the propagation process, and no bacterial colonies were detected before the inoculation.

**Figure 4:**
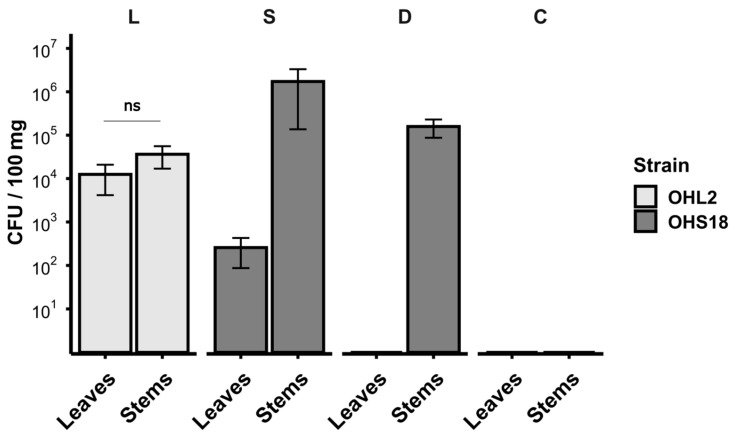
Endophyte colonization after 21 days. Mean viable titer counts (CFU/100 mg) of the endophytic strains *Bacillus* sp. OHL2 and *Pseudomonas* sp. OHS18 obtained for each experimental group (L, S, D, and C) 21 days after the inoculation, in stems and leaves. Error bars represent the standard error. Statistical comparison between leaf and stem tissue was not performed for conditions S and D, where leaf samples showed absent or sporadic bacterial colonization (zero or single non-zero CFU values across biological replicates). For condition L, the Wilcoxon signed-rank test was applied (*p* < 0.05). Statistical significance is indicated as follows: ns, not significant.

Bacterial isolation results (Figure 4) were consistent with those obtained in the previous experiment. Briefly, *Bacillus safensis* OHL2 was recovered from both the stems and the leaves, in each replicate, while *Pseudomonas* sp. OHS18 was isolated almost exclusively from the stems in both S and D experimental groups. In the S condition, OHS18 was detected in leaves in two different replicates; however, its vital titer was four orders of magnitude lower than in the stems (1.9 and 5.8 × 10^2^ CFU/100 mg).

#### 3.4.1. Plant Physiology and Morphology

As *Bacillus safensis* OHL2 was consistently re-isolated from both leaves and stems of axenic plantlets, unlike OHS18, which showed a more restricted tissue distribution—i.e., it was re-isolated basically only from stem tissues-, L-group plants were selected for plant physiology and secondary metabolism analyses, allowing us to verify whether its effects remained specific to leaves, its original niche, despite its presence in both tissues.

**Figure 5:**
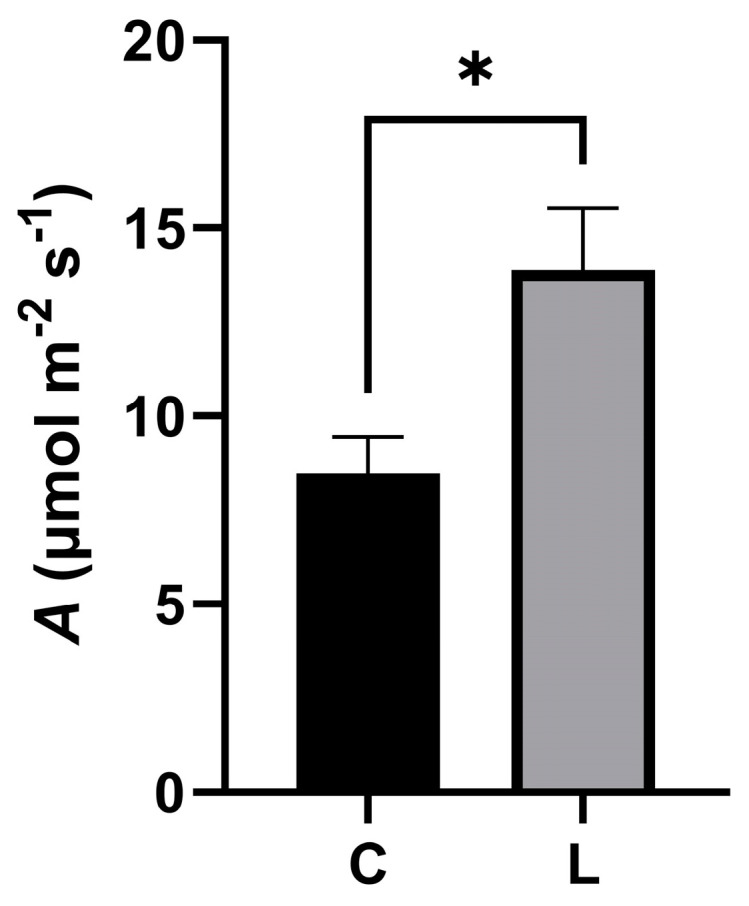
Oregano photosynthetic rate after *Bacillus safensis* OHL2 inoculation. Photosynthetic rate (*A*) of plantlets measured 21 days after inoculation (L) and after the period in control conditions (C). Bars represent means ± standard error (*n* = 5), and asterisks indicate significant differences between treatments at *p* < 0.05 (Unpaired *t* test).

A total of 30 inoculated plantlets belonging to the L group and 30 plantlets belonging to the C group (six plantlets per five replicates for both groups) were analyzed to determine photosynthetic rate (*A*). As shown in Figure 5, *A* was significantly higher in the inoculated plantlets, increasing by approximately 64% compared to the controls.

**Figure 6:**
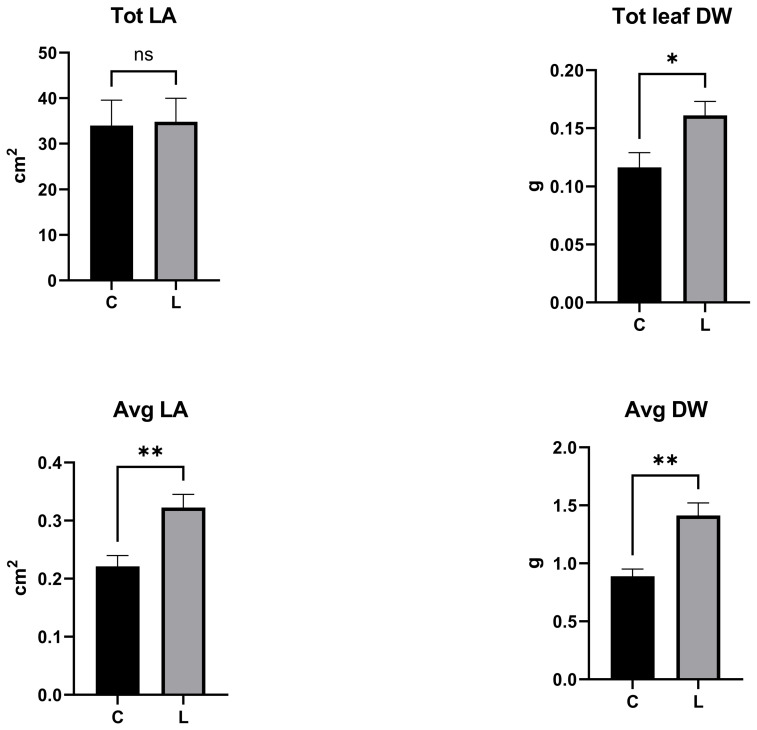
Physiological parameters of oregano plantlets after *Bacillus safensis* OHL2 inoculation. Total leaf area, average leaf area (cm^2^), total leaf dry weight, and average leaf dry weight (g) measured 21 days after inoculation (L) and after the same period in control conditions (C). Values are means ± standard error (*n* = 5), and asterisks indicate significant differences between treatments at *p* < 0.05 (*) or *p* < 0.01 (**); ns, not significant (Unpaired *t* test).

As shown in Figure 6, total leaf area did not change between inoculated and non-inoculated plantlets; however, on average, individual leaves were significantly larger in inoculated plantlets than in controls, indicating a lower leaf number in inoculated plantlets. Total and average leaf dry weight were higher in inoculated plantlets compared with controls.

#### 3.4.2. Secondary Metabolites

**Figure 7:**
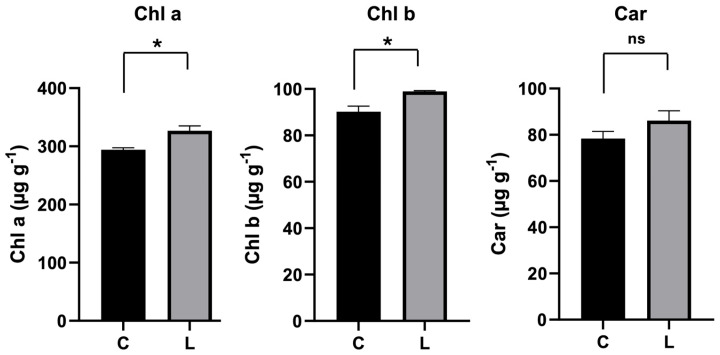
Pigment content of oregano plantlets after *Bacillus safensis* OHL2 inoculation. Chlorophyll a and b (Chl a and Chl b) and carotenoid (Car)concentrations (values expressed as µg of pigments g^−1^ of leaf fresh weight) measured in leaf tissues after 21 days from inoculation (L) and after the same number of days in control conditions (C). Values are means ± standard error (*n* = 3), and asterisks indicate significant differences between treatments at *p*-value < 0.05 (Unpaired *t* test). Statistical significance is indicated as follows: ns, not significant; * *p* < 0.05.

Concerning pigments, a significant increase in the concentration of chlorophyll a and chlorophyll b was assessed in inoculated plantlets compared to controls (Figure 7). On the other hand, carotenoids concentration was not affected by *Bacillus safensis* OHL2 (Figure 7).

Polyphenols analysis of stems and leaves of *O. heracleoticum* showed that rosmarinic acid was the predominant, and essentially the only, polyphenolic compound detected in all extracts. High levels of rosmarinic acid accumulated in both C and L groups (Supplementary Figure S3), reaching approximately 100 and 150 mg g^−1^ DW, respectively, when stems and leaves were considered together.

**Figure 8:**
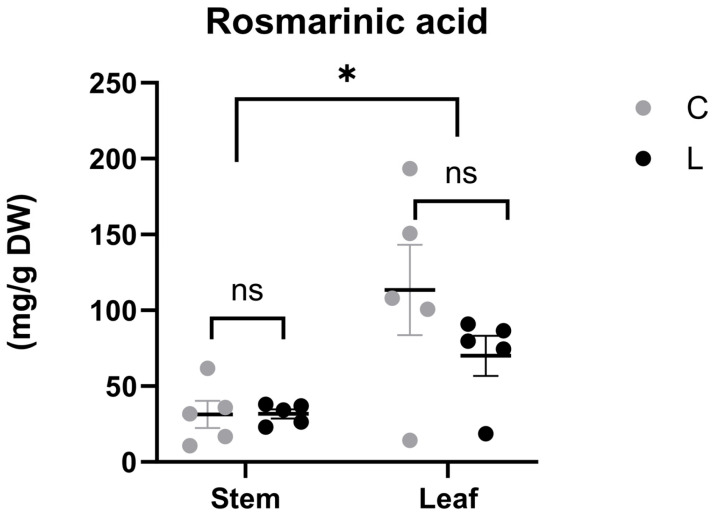
Rosmarinic acid content in leaves and stems after *Bacillus safensis* OHL2 inoculation. Rosmarinic acid concentration (mg g^−1^ of dry weight) was measured in leaf and stem tissues, separately, after 21 days from inoculation (L) and after the same period in control conditions (C). Values are means ± standard mean error (*n* = 5). The mixed effects analysis revealed a significant difference between stems and leaves (*p* < 0.05), no treatment-related effect, nor a significant interaction between the two factors (treatment and tissue). Statistical significance is indicated as follows: ns, not significant; * *p* < 0.05.

When individual compartments were considered (stems or leaves), rosmarinic acid concentration was not affected by inoculation with *Bacillus safensis* OHL2, as no significant differences were observed between tissues of the C and L groups (Figure 8). Stems and leaves showed a significantly different rosmarinic acid content; however, this difference was irrespective of the inoculum.

**Figure 9:**
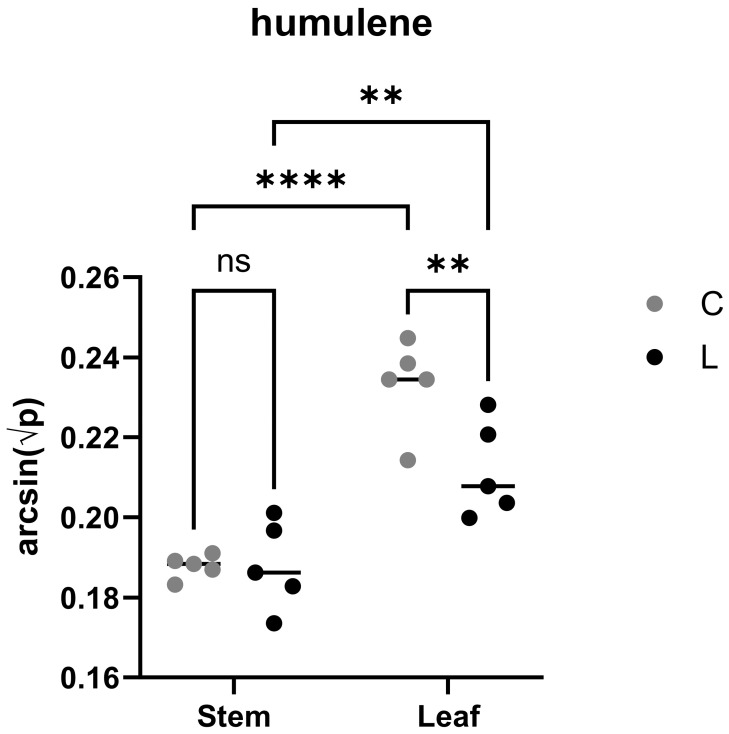
Humulene content in leaves and stems after *Bacillus safensis* OHL2 inoculation. Humulene relative content measured in leaf and stem tissues, separately, after 21 days from inoculation (L) and after the same period in control conditions (C). Values expressed as percentages of the total sesquiterpenes were first converted into proportions (0–1) and then transformed using the arcsine square-root transformation (arcsin(√p), where p = proportion) before statistical analysis. Data are shown as means ± standard mean error (*n* = 5). The mixed effects analysis revealed a significant difference between stem and leaf (*p* < 0.05), a treatment-related effect within the leaf compartment, and a significant interaction between the two factors (treatment and tissue). Statistical significance is indicated as follows: ns, not significant; ** *p* < 0.01; **** *p* < 0.0001.

The terpene profile of *O. heracleoticum* cultivated in vitro was characterized by the presence of 30 terpenes, including five sesquiterpenes (Table S1). Monoterpenes predominated, accounting for approximately 96% of the total identified compounds, with carvacrol as the most abundant constituent, representing about 65% of total monoterpenes. The remaining fraction was constituted by sesquiterpenes (about 4%). Mixed-effects analysis revealed a tissue-related significant difference (*p* < 0.05) for the following compounds: α-Pinene, α-Thujene, Camphene, Sabinene, 3-Carene, β-Myrcene, D-limonene. Additional compounds showing significant tissue-related differences (*p* < 0.05) included γ-Terpinene, p-Cymene, Terpinolene, Linalool, two unidentified terpenes (Unknown terpene 1 and Unknown terpene 2), β-Caryophyllene, Dihydrocarvone isomer, 3-Thujen-2-one, Humulene, Borneol, β-Bisabolene, two unidentified sesquiterpenes (Unknown sesquiterpene 1 and Unknown sesquiterpene 2), and Thymol. For one of the unidentified sesquiterpene 1 (Unknown sesquiterpene 1), a significant effect of inoculation was also detected. In contrast, Humulene showed a significant interaction between tissue and inoculation (Figure 9), with compound levels responding differently to inoculation depending on the tissue considered.

## 4. Discussion

In this work, an efficient in vitro model system of *O. heracleoticum* L. was developed for the study of (i) the interaction(s) between bacterial endophytes and their niche of isolation and (ii) the effects of the inoculation of a selected endophytic strain (*Bacillus safensis* OHL2) on the physiology and secondary metabolism of the axenic plants.

Endophytic strains used in this work were selected from a previously established collection, based on their adaptation to well-defined compartments of *O. heracleoticum* (stems or leaves) [23] and their taxonomic affiliation. Endophytic isolates belonging to the *Bacillus* and *Pseudomonas* genera were frequently identified as plant growth-promoting (PGP) or biocontrol agents [46,47,48,49] and were also found to be able to increase the metabolite content of commercially valuable medicinal and aromatic plants [49,50]. Functional annotation of the genomes of OHL2 and OHS18 strains further supported this rationale, revealing the presence of specific functions associated with an endophytic lifestyle and a putative role as PGP bacteria (Figure S1). To allow the co-inoculation of the two strains, preliminary in vitro tests were performed. The antagonistic interaction between the two endophytes was evaluated through the cross-streaking test in a previous work, revealing no signs of growth inhibition [34]. This finding was further supported by the experiments conducted in a liquid minimal medium, in which the culture filtrate of each strain enhanced the growth of the other, indicating a positive interaction rather than antagonism (Figure S2B). Moreover, to determine whether differential growth rates could confer a competitive advantage during plant colonization, the growth kinetics of the two bacteria in liquid media were obtained. Despite significant differences in the initial growth rate (exponential phase), the overall growth profiles of the two strains were largely comparable under the tested experimental conditions, suggesting that the intrinsic growth capacities of the two strains should not drive colonization outcomes inside the plant (Figure S2A).

Results obtained from the bacterial isolation protocol provided insights into the fate of endophytes within axenically grown *O. heracleoticum* plants, highlighting their niche-specific adaptation and co-evolution within the plant, particularly for *Pseudomonas* sp. OHS18. The preference for a specific niche, or microbiome compartmentalization, has been frequently observed in the study of plant microbiomes [10,34,51,52,53,54,55,56,57]. Accordingly, results obtained for the S and D group strongly suggest the preference of the stem-associated endophyte *Pseudomonas* sp. OHS18 for its niche of origin, as the strain was almost exclusively found in the stem compartment (Figure 3 and Figure 4). Conversely, when *Bacillus safensis* OHL2 was individually inoculated (L group), the strain was detected in both leaves and stems of the axenic model (Figure 3 and Figure 4), at least within the 28-day experimental period. Given that the inoculation of the plantlets was performed via the MS agar medium, the observed data suggest that initial colonization likely occurred through the stem, with a subsequent broader distribution into the leaf tissues, compared to *Pseudomonas* sp. OHS18. Future experiments on the application of the inoculants via other organs (i.e., direct leaf inoculation) can be useful to clarify the actual drivers of *Bacillus safensis* OHL2 tissue colonization.

Concerning the co-inoculation outcomes (D group), the inability to re-isolate *Bacillus safensis* OHL2, despite its successful colonization in single inoculation, hints at the possibility of a key role of the plant itself in regulating bacterial colonization in the endosphere and in shaping competitive interactions [20]. Microbe–microbe interactions within plant habitats are relatively underexplored and may encompass both beneficial relationships and competitive interactions [58]. The pattern observed in our work was not totally unexpected. Studies on in vitro *A. thaliana* models, where individual endogenous strains were introduced into a defined synthetic community, reported that negative interactions are prevalent within plant-associated microbial communities [21]. Therefore, although the two strains showed no antagonistic interaction in vitro, it is plausible that, once inside the plant, *Pseudomonas* sp. OHS18 gained a selective advantage, promoting its own colonization while limiting the progression of *Bacillus safensis* OHL2 within host tissues. Both *Pseudomonas* spp. and *Bacillus* spp. have been found to synthesize a huge repertoire of secondary metabolites with antimicrobial activity [59,60,61]. These antimicrobials are usually produced from gene clusters that remain inactive under standard laboratory or pure culture conditions, but become activated in association with plant microenvironments [62]. Regarding competitiveness between the two genera, available data (reviewed in Lyng and Kovács, 2023) revealed that *Pseudomonas* spp. more often employ competitive traits, while *Bacillus* spp. are generally more defensive [63]. Considering these observations, the inability of *Bacillus safensis* OHL2 to colonize the axenic model when co-inoculated with *Pseudomonas* sp. OHS18 could be the result of the activation of the antibacterial arsenal of the latter strain in the presence of the plant tissues. In particular, the presence of a complete set of genes encoding the bacterial type VI secretion system (T6SS), including structural, regulatory, and effector-associated components (Figure S4), suggests a potential molecular basis for antagonistic interactions. Although we did not assess T6SS expression or activity under our experimental conditions, this system has been shown to provide fitness advantages for bacteria in planta, underlining its specific importance for niche colonization [64,65,66,67,68]. Additionally, bacteria can use indirect antagonistic mechanisms, such as the rapid and efficient utilization of limiting resources (competitive exclusion). The degree of overlap in nutrient utilization among different taxa can influence the outcome of their interaction, determining the extent of their niche differentiation [22,34,58]. Our previous work revealed distinct carbon source preferences and metabolic performances between endophytic strains isolated from the leaf and stem compartments of *O. heracleoticum* [34]. It is therefore plausible that *Pseudomonas* sp. OHS18 showed higher fitness during stem colonization by more efficiently exploiting the available resources, thereby outcompeting *Bacillus safensis* OHL2. In this context, the dominance of *Pseudomonas* sp. OHS18 may reflect a priority effect, whereby early or more efficient establishment within plant tissues determines subsequent exclusion of the second colonizer [69]. Overall, bacterial isolation results allowed us to envision the fate of the two endophytic strains as they move through and colonize the plant tissues, in the absence of a pre-existing cultivable microbiota. Although the presence of non-culturable microorganisms cannot be completely ruled out [70], this approach ensured a higher level of control over plant–microbe interactions and allowed the tracking of the inoculated strains without interference from other cultivable members of the microbiota. Moreover, although the mechanisms employed by microbes to interact within plant tissues seem to play fundamental roles in shaping and structuring microbial networks, the contribution of each type of interaction in the final assembly of the plant-associated bacterial communities remains difficult to evaluate in plants grown under natural conditions due to the complex ecological interactions taking place [20,21,71]. In this regard, the axenic in vitro model described in this work contributes to the understanding of how host–bacteria and bacteria–bacteria interactions in planta shape the establishment of endophytic communities and contribute to their overall assembly, representing a valuable tool to investigate the dynamics of endophyte colonization and distribution.

The inoculation of bacterial endophytes to improve plant performance and commercial value is nowadays a widespread practice in the cultivation of medicinal and aromatic plants [72,73]. PGP bacteria employ a wide range of mechanisms to directly promote plant growth and development, and are known to respond ahead of their host to any environmental perturbation, regulating their gene expression and modulating physiological responses and plant defense-related pathways [74]. In our experimental setting, inoculation of *O. heracleoticum* axenic plants with *Bacillus safensis* OHL2 significantly increased photosynthetic rate, leaf area, dry weight, and chlorophyll content (Figure 5, Figure 6 and Figure 7), key indicators of plant performance and yield [75]. *Bacillus* spp. are widely reported to enhance photosynthetic performance and growth traits, with plant responses generally attributed to PGP traits such as phytohormone biosynthesis and emission of microbial volatile organic compounds [76,77,78,79,80,81,82,83]. However, the effects of bacterial inoculation on plant performance are often mediated indirectly through shifts in microbial community structure [21,84], which ultimately shape plant responses [71]. In this regard, the in vitro axenic system of *O. heracleoticum*, in the absence of a resident cultivable microbial community, provides a valuable tool to disentangle the direct effects of bacterial inoculation. To this end, the molecular and phenotypic characterization of *Bacillus safensis* OHL2 PGP traits, together with an investigation of the molecular basis underlying the plant responses, will further elucidate the mechanism by which the endophyte interacts with the plant and whether the produced effects are specifically tied to its organ of origin.

Endophytes residing in secondary metabolites-producing tissues may also act as inducers of plant metabolic capabilities, either by assisting in the production of these compounds or by transforming precursor molecules into novel metabolites [85,86]. The main, and essentially the only, polyphenol present in the extracts of *O. heracleoticum* was rosmarinic acid. This is consistent with previous results regarding the phenolic composition of the plant, also known as Greek oregano, where rosmarinic acid was the only component found in ethanolic extracts (about 1271 mg per 100 g of plant extract), except for traces of caffeic acid [87]. Rosmarinic acid possesses various relevant biological activities, and it is produced by plants as a defense compound [88,89]. The accumulation of such a high level of rosmarinic acid observed in both C and L groups (Figure 8) can be attributed to the in vitro cultivation of *O. heracleoticum* [90,91]. In fact, this hydroxycinnamic acid largely accumulates in species of the *Lamiaceae* family during in vitro cultures, reaching up to 36% of dry weight, as in the case of *Salvia* spp. [92,93,94]. In vitro culture can be considered a successful technique for large-scale production of hydroxycinnamic acid derivatives, which can be employed in nutraceutical and pharmaceutical applications thanks to their high antioxidant properties [95,96]. Moreover, the possibility of using a micropropagated clonal line reduces the individual variability among plantlets within a certain phenotype, allowing a precise comparison among replicates.

Considering the terpene profile of the in vitro cultivated *O. heracleoticum* plants, monoterpenes were predominant, with carvacrol as the most abundant compound (Table S1). Terpene production in in vitro–grown *Origanum* spp. has already been demonstrated, with studies reporting the accumulation of carvacrol and related monoterpenes in micropropagated plants and callus cultures [97,98]. The results are also consistent with the chemical characterization of the essential oil of *O. heracleoticum* plants from which the two endophytes were originally isolated, where carvacrol accounted for 73.9% of the total volatile blend [23]. Concerning sesquiterpenes, humulene showed a significant interaction between tissue type and bacterial inoculum, with reduced levels in the leaves of inoculated plants compared to the control (Figure 9). These results suggest the ability of *Bacillus safensis* OHL2 to modulate plant secondary metabolism, specifically affecting its tissue of origin. Plants produce humulene as part of their complex sesquiterpenoid defense and communication system, with repellent, antifeedant, and antimicrobial properties [99]. Li et al. (2024) demonstrated that limited nitrogen availability enhanced tomato resistance against the agricultural pest *Spodoptera litura*, a response that was associated with the increased biosynthesis and emission of α-humulene [100]. This is consistent with the adaptive growth hypothesis: since both growth and defense mechanisms are resource- and energy-consuming, plants dynamically distribute metabolic investments based on their nutritional status [101]. In the present study, the inoculation with *Bacillus safensis* OHL2 increased plant biomass and enhanced photosynthetic performance, consistent with a possible reallocation of plant resources toward growth-related processes. However, the underlying mechanisms remain unclear, and alternative explanations, including indirect effects mediated by plant–microbe interactions, cannot be excluded. Future transcriptomic analyses will be required to disentangle the plant- and bacterium-driven components of this interaction and to elucidate the ecological role of the selected strain [47,102].

## 5. Conclusions

Data obtained in this work further indicate a non-random distribution of bacterial strains between leaves and stems of *O. heracleoticum*, shaped by plant microenvironments and microbe–microbe interactions in planta. The in vitro plant model was deliberately designed as a simplified system to disentangle the specific bacterial mechanisms involved in stimulating plant metabolism and physiology from the complexity of field conditions, where colonization is influenced by the full plant microbiome and environmental factors. While we acknowledge that this controlled system does not capture the full intricacy of natural plant–endophyte interactions, it provides a tractable framework to further investigate colonization dynamics in *Origanum* species. Furthermore, the ability to apply bacterial inoculants individually presents an opportunity to identify promising strains for biotechnological applications: the understanding of how bacterial endophytes adapt to and influence specific plant compartments may lead to the development of strategies to manipulate these microbial communities in ways that enhance plant growth, improve secondary metabolite yields, or even produce novel compounds through microbial transformation. This is of tremendous interest for medicinal and aromatic plants, where secondary metabolism could be enhanced or modulated by their endophytic microbial resources.

## Figures and Tables

**Table 1:** Bacterial suspensions (OD_600_ = 0.01) used in each experimental condition.

Condition	Bacterial Strains	Vital Titer Count (CFU/mL)
L	*Bacillus* sp. OHL2	4.1 × 10^6^
S	*Pseudomonas* sp. OHS18	2.6 × 10^6^
D	*Bacillus* sp. OHL2 + *Pseudomonas* sp. OHS18	4.0 × 10^6^ 2.8 × 10^6^
C	Saline solution	Ø

## Data Availability

The original contributions presented in this study are included in the article/Supplementary Materials. Further inquiries can be directed to the corresponding authors.

## Supplementary Materials

The following supporting information can be downloaded at: https://www.mdpi.com/article/10.3390/microorganisms14071497/s1, Figure S1: Heatmap representation of the frequency of plant growth-promoting traits identified through functional annotation. The annotation of bacterial plant growth-promoting traits was performed using the blastp + hmmer (strict mode) mapping against the PGPT Ontology. The eight groups at Level 2, and 43 subcategories at Level 3 of the annotation were used for heatmap construction; Figure S2: (A) Growth curves of the two bacterial strains. Points show mean OD_600_ at each time point (1 h), with error bars representing standard error (B) Growth of bacterial strains OHL2 and OHS18 in minimal medium in the presence or absence of culture filtrate from the other strain. Bars represent the mean OD_600_ values measured after 2 and 5 days, and error bars indicate the standard error. Letters above bars indicate statistically significant differences between conditions (treatment vs. control) at each time point (*p* < 0.05); Figure S3: Rosmarinic acid concentration (mg/g of dry weight) measured in leaf and stem tissues after 21 days from inoculation (L) and after the same number of days in control conditions (C). Values are means ± standard mean error (n = 5); Table S1: Terpene contents of *O. heracleoticum* in vitro plants. Values represent means ± standard deviation; Figure S4: Type VI secretion system (T6SS) gene cluster annotated in the *Pseudomonas* sp. OHS18 genome using BLASTp and HMMER (strict mode) mapping against the PGPT Ontology, performed with the PGPT-Pred tool available on the PLaBAse server. The genomic organization of the annotated genes was visualized in R using the ‘gggenes’ and ‘ggplot2’ packages.
