# Supplementary material for: Plant–Endophyte Cross-Talk in Origanum heracleoticum L. In Vitro Axenic Culture: Endosphere-Driven Bacterial Interactions and Plant Metabolic Responses
Source: Microorganisms. 2026 Jul 8;14(7):1497. doi: 10.3390/microorganisms14071497 (PMC13413973; doi:10.3390/microorganisms14071497)
Supplement: Supplementary file 1 [file microorganisms-14-01497-s001.zip › microorganisms-4369240-supplementary.pdf]

Supplementary Materials

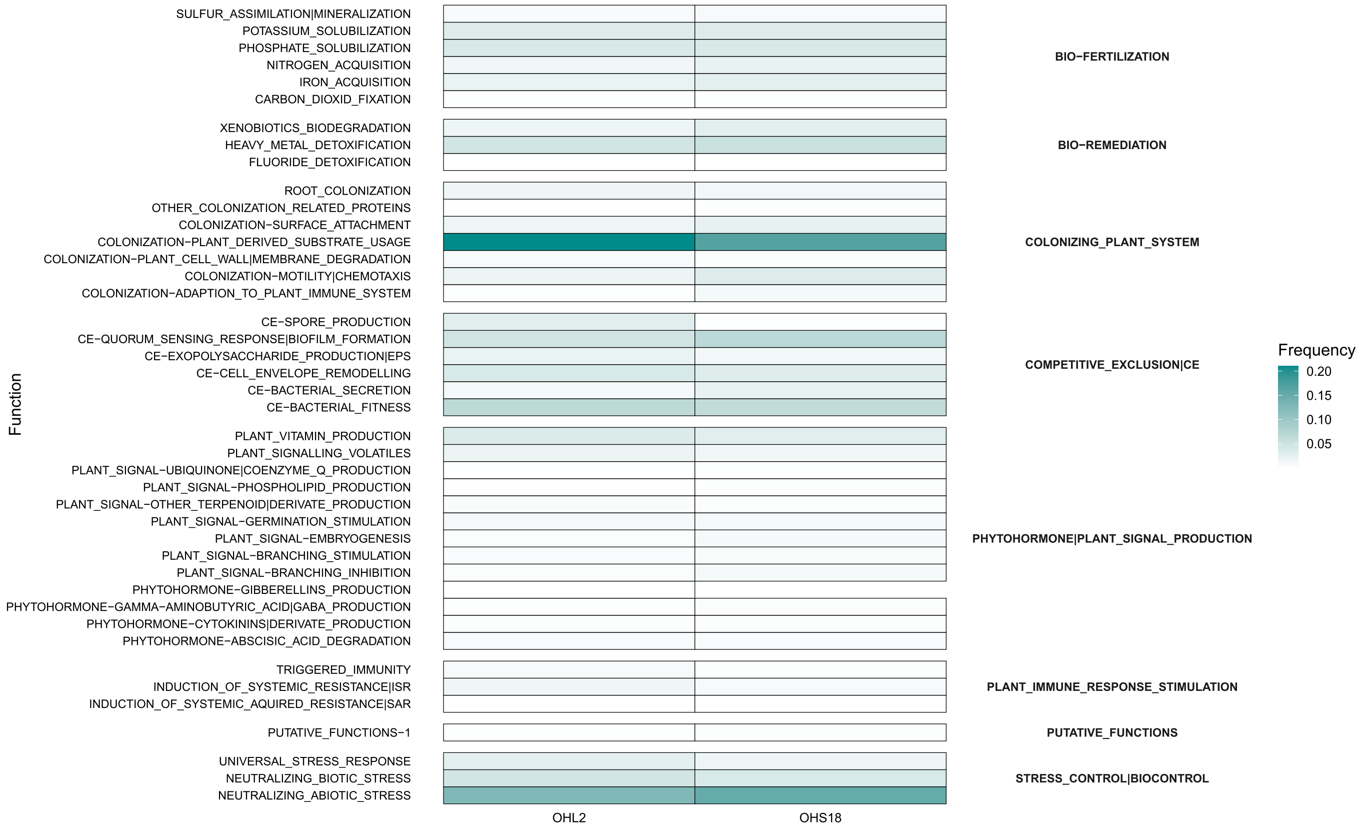

Figure S1. Heatmap representation of the frequency of plant growth-promoting traits identified through functional annotation. The annotation of bacterial plant growth-promoting traits was performed using the blastp + hmmer (strict mode) mapping against the PGPT Ontology. The eight groups at Level 2, and 43 subcategories at Level 3 of the annotation were used for heatmap construction.

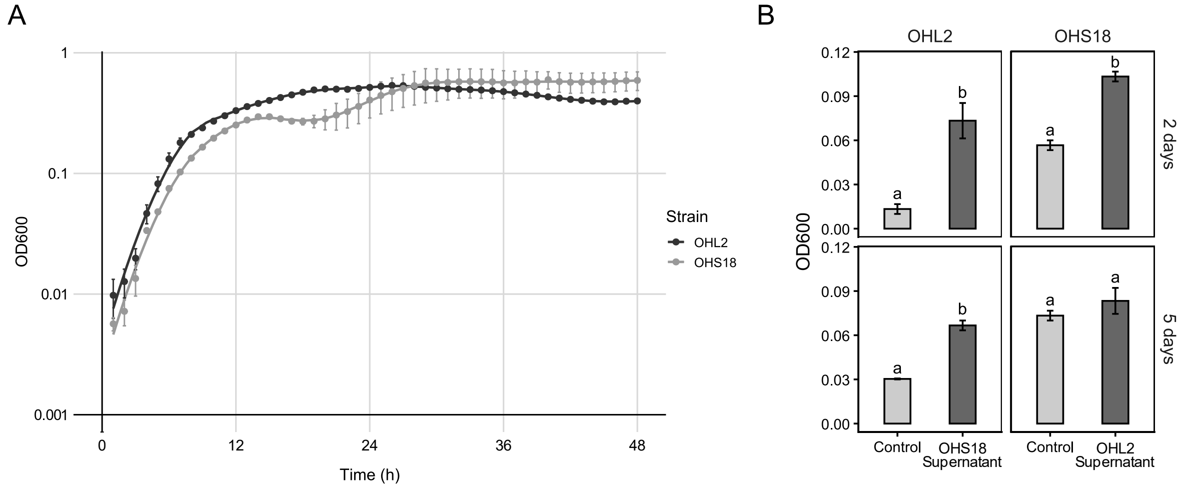

Figure S2. (**A**) Growth curves of the two bacterial strains. Points show mean OD600 at each time point (1 h), with error bars representing standard error (**B**) Growth of bacterial strains OHL2 and OHS18 in minimal medium in the presence or absence of culture filtrate from the other strain. Bars represent the mean OD600 values measured after 2 and 5 days, and error bars indicate the standard error. Letters above bars indicate statistically significant differences between conditions (treatment vs. control) at each time point ($p < 0.05$).

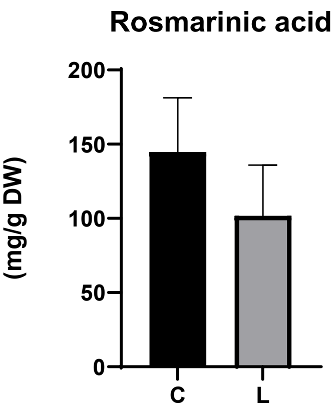

Figure S3. Rosmarinic acid concentration (mg/g of dry weight) measured in leaf and stem tissues after 21 days from inoculation (L) and after the same number of days in control conditions (C). Values are means ± standard mean error (n = 5).

Table S1. Terpene contents of *O. heracleoticum in vitro* plants. Values represent means ± standard deviation.

	Stem C	Leaf C	Stem L	Leaf L
α-Pinene	1.671 ± 0.257	1.439 ± 0.190	1.759 ± 0.380	1.392 ± 0.087
α-Thujene	1.866 ± 0.177	3.319 ± 0.536	1.877 ± 0.215	3.271 ± 0.138
Camphene	2.230 ± 0.304	0.702 ± 0.047	2.270 ± 0.278	0.710 ± 0.098
β-Pinene	0.258 ± 0.035	0.304 ± 0.041	0.291 ± 0.090	0.296 ± 0.013
Sabinene	0.290 ± 0.075	0.459 ± 0.064	0.273 ± 0.032	0.446 ± 0.017
3-Carene	0.159 ± 0.076	0.216 ± 0.020	0.138 ± 0.021	0.220 ± 0.022
β-Myrcene	1.891 ± 0.142	3.326 ± 0.510	2.021 ± 0.436	3.212 ± 0.132
α-Phellandrene	0.230 ± 0.028	0.235 ± 0.044	0.227 ± 0.033	0.204 ± 0.018
α-Terpinene	1.459 ± 0.330	1.527 ± 0.252	1.328 ± 0.272	1.423 ± 0.262
D-Limonene	0.347 ± 0.143	0.320 ± 0.036	0.357 ± 0.155	0.296 ± 0.022
β-Phellandrene	0.310 ± 0.063	0.364 ± 0.049	0.366 ± 0.276	0.343 ± 0.015
trans-β-Ocimene	0.569 ± 0.108	0.536 ± 0.159	0.553 ± 0.307	0.486 ± 0.080
γ-Terpinene	9.227 ± 3.301	11.602 ± 1.771	8.814 ± 1.491	12.368 ± 0.602
p-Cymene	4.852 ± 1.412	6.758 ± 0.934	4.702 ± 0.558	7.069 ± 1.110
Terpinolene	0.245 ± 0.026	0.129 ± 0.006	0.253 ± 0.059	0.123 ± 0.015
Linalolo	0.491 ± 0.075	0.289 ± 0.044	0.483 ± 0.036	0.309 ± 0.020
Unk terpene 1	0.348 ± 0.043	0.401 ± 0.027	0.350 ± 0.019	0.401 ± 0.023
Unk terpene 2	0.063 ± 0.009	0.152 ± 0.011	0.068 ± 0.011	0.147 ± 0.017
Dihydrocarvone	0.223 ± 0.029	0.228 ± 0.015	0.216 ± 0.024	0.219 ± 0.017
Dihydrocarvone isomer	0.112 ± 0.015	0.218 ± 0.021	0.115 ± 0.038	0.221 ± 0.028
3-Thujen-2-one	0.087 ± 0.029	0.152 ± 0.025	0.091 ± 0.027	0.160 ± 0.020
α-Terpineol	0.137 ± 0.063	0.186 ± 0.034	0.126 ± 0.024	0.146 ± 0.038
Borneol	6.431 ± 0.798	2.024 ± 0.192	6.447 ± 0.495	2.030 ± 0.226
Thymol	0.438 ± 0.144	0.659 ± 0.142	0.539 ± 0.148	0.740 ± 0.057
Carvacrol	66.065 ± 1.611	64.454 ± 2.944	66.336 ± 4.077	63.766 ± 1.215
**Total monoterpenes**	**95.090 ± 0.296**	**96.113 ± 0.501**	**95.179 ± 0.629**	**96.149 ± 0.283**
β-Caryophyllene	20.748 ± 1.319	26.834 ± 0.939	20.494 ± 2.555	26.725 ± 1.017
α-Humulene	3.484 ± 0.108	5.355 ± 0.504	3.504 ± 0.405	4.439 ± 0.497
β-Bisabolene	67.854 ± 1.909	54.057 ± 2.546	68.852 ± 2.736	55.755 ± 1.890
Unk sesqui1	3.450 ± 0.377	6.927 ± 1.739	2.091 ± 0.679	5.913 ± 0.574
Unk sesqui2	4.464 ± 1.541	6.826 ± 1.245	5.058 ± 1.940	7.168 ± 1.105
**Total Sesquiterpenes**	**4.910 ± 0.296**	**3.887 ± 0.501**	**4.821 ± 0.629**	**3.851 ± 0.283**

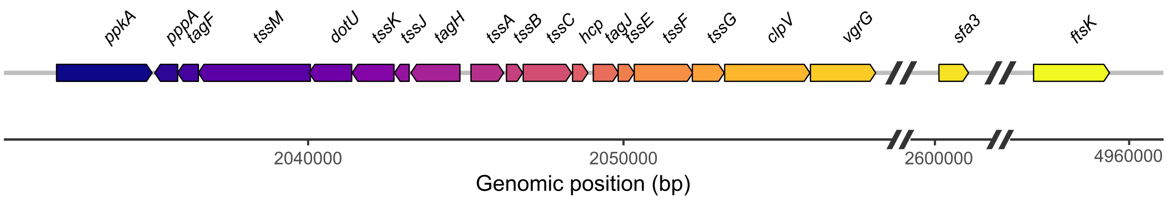

Figure S4. Type VI secretion system (T6SS) gene cluster annotated in the *Pseudomonas* sp. OHS18 genome using BLASTp and HMMER (strict mode) mapping against the PGPT Ontology, performed with the PGPT-Pred tool available on the PLaBAse server. The genomic organization of the annotated genes was visualized in R using the '*gggenes'* and '*ggplot2'* packages.

Gene functions are indicated as follows: ***clpV***: type VI secretion system ATPase; ***dotU***: DotU family type secretion system protein; ***ftsK***: DNA traslocase; ***hcp***: Hcp family type VI secretion effector; ***pppA***: PP2C family protein Ser/Thr hosphatase; ***ppkA***: protein kinase domain-containing protein; ***sfa3***: sigma 54 – interacting transcriptional regulator; ***tagF***: type VI secretion system associated protein TagF; ***tagH***: type VI secretion system associated FHA domain protein TagH; ***tagJ***: type VI secretion system accessory protein TagJ; ***tssA***: type VI secretion system protein TssA; ***tssB***: type VI secretion system contractile sheath small subunit; ***tssC***: type VI secretion system contractile sheath large subunit; ***tssE***: type VI secretion system baseplate subunit TssE; ***tssF***: type VI secretion system baseplate subunit TssF; ***tssG***: type VI secretion system baseplate subunit TssG; ***tssJ***: type VI secretion system lipoprotein TssJ; ***tssK***: type VI secretion system baseplate subunit TssK; ***tssM***: type VI secretion system membrane subunit; ***vgrG***: type VI secretion system tip protein TssI/VgrG.
